# Investigation of Hydrogen Embrittlement Susceptibility and Fracture Toughness Drop after in situ Hydrogen Cathodic Charging for an X65 Pipeline Steel

**DOI:** 10.3390/mi11040430

**Published:** 2020-04-20

**Authors:** Helen P. Kyriakopoulou, Panagiotis Karmiris-Obratański, Athanasios S. Tazedakis, Nikoalos M. Daniolos, Efthymios C. Dourdounis, Dimitrios E. Manolakos, Dimitrios Pantelis

**Affiliations:** 1 Shipbuilding Technology Laboratory, School of Naval Architecture and Marine Engineering, National Technical University of Athens, 15780 Athens, Greece; 2 School of Mechanical Engineering and Robotics, AGH University of Science and Technology, Mickiewicza 30, 30-059 Cracow, Poland; pkarm@mail.ntua.gr; 3 School of Mechanical Engineering, National Technical University of Athens, Heroon Polytechniou 9, 15780 Athens, Greece; manolako@central.ntua.gr; 4 Corinth Pipeworks S.A., Industrial Area of Voiotia, Thisvi, 32010 Domvraina, Greece; atazedakis@cpw.vionet.gr (A.S.T.); ndaniolos@cpw.vionet.gr (N.M.D.); edourdounis@cpw.vionet.gr (E.C.D.)

**Keywords:** X65 pipeline steel, hydrogen embrittlement, in situ hydrogen cathodic charging, crack tip opening displacement test (CTOD), crack mouth open displacement (CMOD), fracture analysis

## Abstract

The present research focuses on the investigation of an in situ hydrogen charging effect during Crack Tip Opening Displacement testing (CTOD) on the fracture toughness properties of X65 pipeline steel. This grade of steel belongs to the broader category of High Strength Low Alloy Steels (HSLA), and its microstructure consists of equiaxed ferritic and bainitic grains with a low volume fraction of degenerated pearlite islands. The studied X65 steel specimens were extracted from pipes with 19.15 mm wall thickness. The fracture toughness parameters were determined after imposing the fatigue pre-cracked specimens on air, on a specific electrolytic cell under a slow strain rate bending loading (according to ASTM G147-98, BS7448, and ISO12135 standards). Concerning the results of this study, in the first phase the hydrogen cations’ penetration depth, the diffusion coefficient of molecular and atomic hydrogen, and the surficial density of blisters were determined. Next, the characteristic parameters related to fracture toughness (such as J, KQ, CTOD_el_, CTOD_pl_) were calculated by the aid of the Force-Crack Mouth Open Displacement curves and the relevant analytical equations.

## 1. Introduction

Over the last 15 years, the main targeting of the petroleum and hydrocarbon industry has been to encounter the phenomenon of hydrogen embrittlement and hydrogen induced toughness drop of the oil carrying pipeline steels during their operational cycle [1,2,3,4,5] The presence of molecular H_2_S in the internal environment of pipeline steels, attributed to the oil decreased quality, results in the diffusion and entrapment of atomic hydrogen into metal structure, which leads to the reinforcement of Hydrogen Induced Cracking (HIC) phenomenon, blistering effect and development of brittle hydride phases (FeH_3_) [5,6,7,8,9].The surface micro-cracking networks have a characteristic stepwise morphology, whereas blisters depending on their growth conditions may occur locally with a dome morphology or in an extended area with elongated and continuous morphology (parallel to the thermoplastic deformation axis) [9,10,11,12]. The blisters grow on lattice defects, where the hydrogen cations accumulate continuously. These microstructural sites are separated into irreversible or reversible traps, according to the energy needed thermodynamically for the hydrogen release process. Irreversible traps include vacancies, high angle grain boundaries, dislocation forests and non-metallic inclusions, while reversible traps include interstitial lattice sites, internal or external stacking faults, low angle grain boundaries and micro-twin boundaries [12,13,14]. Finally, the development of FeH_3_ hydrides was detected to occur mainly at energy upgraded locations such as the interfaces between non-metallic inclusions and matrix as well as the triple junctions. 

In particular, three micro mechanisms were proposed in the scientific field of physical metallurgy to justify the reduction of mechanical properties on pipeline steels due to the effect of atomic or molecular hydrogen, reported as Internal Pressure theory (IP), Hydrogen Enhanced Local Plasticity theory (HELP) and Hydrogen Induced Decohesion theory (HEDE).

The first relates to the development of elevated internal pressures due to hydrogen adsorption on the micro-cavities of the microstructure, the second to the local increase of the microplasticity resulting in the excess of yield point and the third to the reduction of the interconnectivity between grain boundaries and boundaries of different phases and microstructural components [15,16,17,18,19,20,21].

The hydrogen embrittlement phenomenon leads to the deterioration of the chemico-mechanical integrity of the main metallurgical systems in the contemporary technological field. Its destructive effect was observed in alloy systems with Face Centered Cubic (FCC), Body Centered Cubic (BCC), and Hexagonal Close Packed (HCP) structures and is particularly pronounced in high strength low alloyed steels. The underlying mechanisms which contribute to this phenomenon are still not fully understood, despite the extensive research efforts in the oil and hydrogen gas pipeline transportation industry.

For the pipeline steels the proposed embrittling micromechanisms mainly concern the formation of hydride phases, the reduction of the interfacial cohesive strength within the crystallographic structure due to the mitigation effect of hydrogen atoms (HEDE) and the hydrogen enhanced localized plasticity phenomenon (HELP). Hydride formation is favored by the presence of residual stress fields and is very common in metallurgical systems that exhibit a thermodynamic and kinematic tendency for hydride formation.

During the evolution of Hydrogen Enhanced Decohesion mechanism (HEDE), the hydrogen cations accumulate within the high and low energy trapping centers of the crystal structure, reducing the cohesive energy strength within the grains of the matrix. This results in a severe reduction of the mechanical energy required to decode the crystallographic structure along certain crystallographic planes. As the more prone regions are considered the grain boundaries with high angle misorientation distribution factor and the interphase boundaries with elevated dislocation densities. 

The ductile surface of many metallurgical systems that had failed due to the penetration effect of hydrogen cations led to the support of a newly developed mechanism, named Hydrogen Enhanced Localized Plasticity (HELP). According to this mechanism, the propagation rate of dislocation networks and the dislocation core energy increases in areas with elevated concentrations of hydrogen cations, such as the micro-cracked and notched regions. Consequently, a significant increase in local plasticity is observed, which results in a high surficial density of shear slip bands and Ludders’ bands causing the overall failure of the structure. In recent published work M. B. Djukic, et al. investigated the hydrogen embrittlement susceptibility of a low carbon structural steel (grade 20-St.20 (GOST 1050-88)). This grade of steel is used for the construction of boiler tubes of fossil fuel power plants and is subjected to high temperature hydrogen attack and degradation of the mechanical properties due to hydrogen embrittlement process. Based on the experimental results the contribution of hydrogen enhanced decohesion (HEDE) and hydrogen enhanced localized plasticity (HELP) mechanisms is detected, depending on the local concentration of hydrogen cations, measured by means of Thermal Desorption Spectroscopy. The coexistence of the main embrittlement mechanisms (HELP+HEDE) is confirmed by a mixed quasi-cleavage mode of fracture with the presence of brittle transgranular fracture surfaces into the ferrite islands (activity of HEDE mechanism) and microvoid coalescence fracture morphologies into the pearlitic microconstituents (activity of HELP mechanism) [22]. Rajwinder Singh et al. investigated the hydrogen influenced crack initiation and propagation under tensile and low cycle fatigue loadings in RPV steel. It is detected that under low cycle fatigue loading of the uncharged specimen, multiple microcrack networks initiated at the fractured surfaces from M_3_C carbides distributed along the bainitic ferrite lath boundaries. The microcracks initiated also from the non-coherent interphases between angular Al_2_O_3_-SiO_2_ inclusions and matrix material. In contrast for the specimens subjected to low cycle fatigue under hydrogen cathodic charging process the edges of cracks formed on the surface of the specimens during the first stage of LCF loading and propagated throughout the cross section with transgranular and intergranular fracture mode due to the evolution of HEDE mechanism. For the uncharged specimens a slip driven /transgranular fatigue crack propagation mode was detected counter to the hydrogen charged specimens, where the propagation of fracture was developed by the contribution of an intergranularly and transgranularly mode. Due to the hydrogen-impeded localized plasticity no striations are observed on the fractured surfaces of hydrogen charged specimens. In conclusion, different hydrogen embrittlement mechanisms were activated throughout the thickness of the specimens depending on the local hydrogen concentration level and the loading conditions [23]. M.B. Djukic, et al. investigated the hydrogen damage phenomenon in steels and proposed a hydrogen embrittlement model. The coexistence of HELP and HEDE embrittlement mechanisms was detected by the mixed mode of fracture by quasi-cleavage regions (QC), transgranular fracture features in ferrite islands (TG) and the evolution of microvoid coalescence phenomenon (MVC). For the lower hydrogen concentrations, the dominant fracture mechanism is considered to be HELP, due to the increased volume fraction of ductile MVC fracture features in the “quasi-cleavage” fracture surfaces of ferritic islands. For critical values of hydrogen concentration into the metal structure an expanded manifestation of HEDE mechanism is determined by sudden transition between ductile and brittle fracture modes. The restriction of HELP mechanism leads to the ductility drop and enhances the activity of HEDE mechanism by the severe reduction of cohesive strength between the interfacial grain boundaries. Finally, fracture toughness and microhardness values can be successfully correlated with the hydrogen cations’ concentration factor in microstructural sites. The fracture toughness of steel decreases with an increase of hydrogen concentration factor, regardless the type of the existing embrittlement mechanism [17,18,19,20,21,24].

Specifically for X52, X60, X65, X70, X80, and X100 pipeline steels it was found that during their operation in sour environment a significant decrease in ductility, maximum tensile strength, and fracture toughness parameters (K_Q_, K_IC_, J_el_, J_pl,_ CTOD_el_, CTOD_pl_) takes place in relation to the adsorbed concentration of atomic or molecular hydrogen. Experimentally, the above phenomenon is studied either by ex situ or in situ hydrogen cathodic charging process during the Crack Tip Opening Displacement (CTOD) test, with selected parameters simulating the actual operating conditions. Particularly important has been proving the electrolyte composition (hydrogen content), the applied current density and the duration of the cathodic charging process. Specifically for the in situ hydrogen cathodic charging method, a higher decrease in toughness is observed primarily with increasing the applied current density, secondary with increasing the hydrogen content in the electrolytic environment and third with increasing the hydrogen cathodic charging duration.

The volume fraction, the morphology and the distribution of non-metallic inclusions, carbides, and Martensite-Austenite islands (MAs), the dislocation density as well as the existence of a ferritic-pearlitic or a ferritic-bainitic or a mixed ferritic-pearlitic-bainitic microstructure in pipeline steels are the key factors that influence the Hydrogen Induced Cracking resistance performance. Chatzidouros et al. studied the toughness drop after the in situ hydrogen cathodic charging process during CTOD testing for API5L X52, X65 and X70 steels, with a ferritic-pearlitic, ferritic-bainitic and ferritic-mixed pearlitic-bainitic microstructure respectively [25,26]. The X65 pipeline steel was proved to be more prone to the reduction of J_o_ and CTOD toughness parameters due to the adsorbed hydrogen concentration in the lattice structure than the other two grades of pipeline steels. For the maximum value of the applied current density field (10 mA/cm^2^) a severe decrease of J_o_ toughness parameter was detected, which was calculated equal to 77.7%. For the less prone microstructures of X52 and X70 pipeline steels to the hydrogen embrittlement phenomenon, it was observed that the ferritic islands constituted the hydrogen diffusion channels and the ferrite-pearlite or ferrite-bainite interfaces conducted as the main hydrogen trapping centers. 

Through this study, interesting results were provided concerning the hydrogen induced toughness decrease, the micromechanisms which contribute to failure, the morphology and surficial density of blisters and microcracks, the microhardness increment at pipeline steel surfaces, as far the diffusion depth and the diffusion coefficient of hydrogen cations into the structure correlated with the applied current density during the in situ hydrogen cathodic charging process. The novelty of this paper is attributed to the use of an environmental friendly electrolyte consisted of 30 g/L NaCl and 3 g/L NH_4_SCN (which satisfies green chemistry’s requirements) for elevated values of the applied current density field (10 and 20 mA/cm^2^) during the in situ hydrogen cathodic charging process and slow strain rate bending. The applied current densities exceeded the above of other researchers for X65 pipeline steel, which haven’ t been higher than 10 mA/cm^2^. These experiments took place in order to investigate the existence or not of a thermodynamic or electrokinetic potential threshold which leads to the hydrogen absorbed diminishing effect independently of the increment of the applied current density. According to the obtained experimental results, by increasing the applied current density field during in situ hydrogen cathodic polarization process the fracture toughness was decreasing continuously.

## 2. Experimental Section

**Figure 1:**
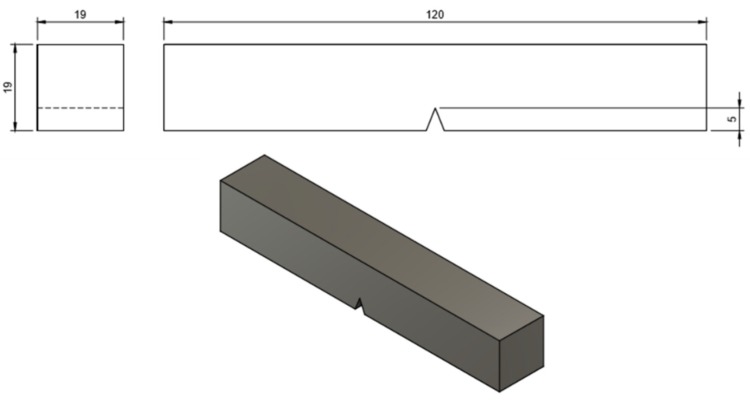
Dimensions of the crack tip opening displacement test (CTOD) specimen.

**Figure 2:**
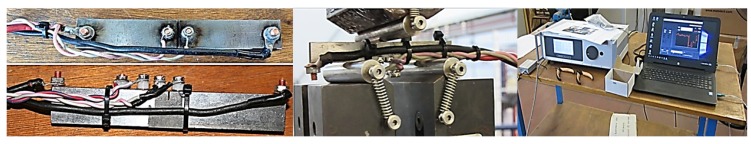
CTOD testing by the applicability of a fatigue pre-crack stage to the notched specimens on air environment.

The dimensions of the crack tip opening displacement test (CTOD) specimen are shown in Figure 1. The Crack Tip Opening Displacement test took place in two separate stages. In the first stage, the controlled crack propagation rate during fatigue loading in air environment was realized (fatigue pre-crack) for the X65 pre-notched specimens. At this stage, the crack length was monitored using an Alternative Pulse Current Device (ACPD). As the fatigue crack propagated, the continuous variation of the electric voltage field and the resistance drop were measured. By means of a calibrated transfer function, the gradient of the electric voltage field and the resistance drop were transformed into fatigue crack propagation length (Figure 2). 

The pre-crack fatigue process of the specimens was carried out with a frequency of 10 Hz, while the parameters of the maximum-minimum load and the cycles are mentioned in Table 1. The different stages during the fatigue precrack process were developed in order to avoid the undesirable effects of plasticity and crack blunting.

In any case, when the crack length reached the critical value of 4 mm, the loading mode transformed to slow strain rate bending until fracture (1.6 × 10^−11^ s^−1^), after the specimen was placed in the specific electrolytic cell.

At this final stage of slow strain rate bending loading, a bimetallic plate, with especially adapted crack open displacement gauges (COD gauges), was applied on the surface of the notched area, so as to record the expansion of the upper lip of the notch. As fracture was completed, by the use of the Force-Crack Mouth Open Displacement (F-CMOD) curves and the analytical fracture mechanic relationships were calculated the toughness parameters *K*_Q_, *J*_m_, CTOD_el_, CTOD_pl_.

**Figure 3:**
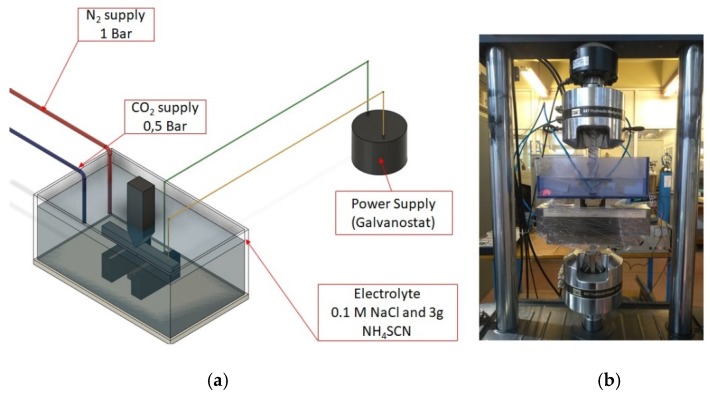
(**a**) Schematic illustration of the in situ hydrogen cathodic charging device, (**b**) Second stage of CTOD testing by the applicability of slow strain rate bending loading under in situ hydrogen cathodic charging conditions (polarization process).

More precisely, in the electrolytic cell during the electrochemical charging process, as cathode was imposed the pre-notched CTOD specimen of X65 steel and as anode was selected a platinum coated titanium electrode. The electrolytic solution used in the cathodic polarization process was consisted of 0.2 M NaCl and 3 g/L NH_4_SCN (which reacts as a poison recombination inhibitor of hydrogen cations on the metal surface). The electrochemical system was stirred by a mixture of CO_2_ and N_2_ gases in order to stabilize the pH value between 5 and 5.2 and promote its electrochemical activity. During the in situ hydrogen cathodic charging process the applied current density was stabilized at 10 and 20 mA/cm^2^, for a cathodic polarization duration of 26 h (according to Petroleum Industry Standard ISO 3183) (Figure 3). The surface of the cathodically charged specimens was insulated with polyurethane, except of the notched area, in order to promote the hydrogen cation electrodiffusion process specifically in this area.

## 3. Results and Discussion

**Figure 4:**
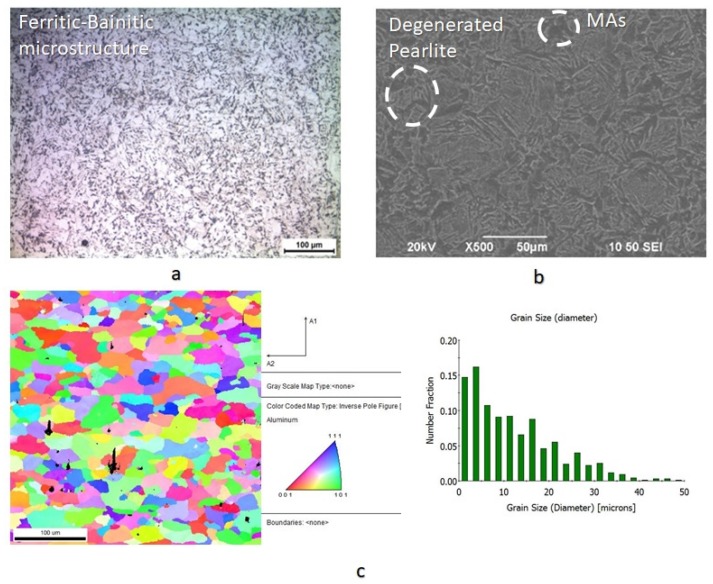
(**a**) Ferritic-Banitic microstructure of X65 pipeline steel by means of Optical Microscopy, (**b**) Degenerated perlite islands and MAs micro/constituency within the crystalographyc and (**c**) grain orientaion mapping and averange grain size by EBSD analysis.

The microstructure of X65 pipeline steel consisted of ferritic and bainitic grains with high volume volume fraction of degenerated pearlite islands. It was also detected the existence of limited proportion of non-metallic inclusions (manganese sulfide, aluminum oxide) and embrittling Martensite-Austenite phases (MAs). The average grain size of the ferritic-bainitic microstructure was evaluated by means of Electron Backscatter Diffraction technique (EBSD) equal to 18 μm (Figure 4). Τhe chemical composition and mechanical properties of X65 steel are shown in Table 2 and Table 3. The studied specimens had dimensions 120 × 19 × 19 mm and had been extracted from pipelines with external diameter 29 in (736.6 mm). These pipelines emanated from the last stage of thermo-mechanical production process without being subjected to sour service conditions. The X65 pipeline steel was developed by a continuous casting process with the imposition of de-oxidation mechanism of Al-killed during industrial refining process.

The effect of the microstructure on the hydrogen cations’ migration phenomenon and microcrack propagation velocity is mainly attribute to the existence of the lower bainite islands and the interfaces between the ferritic-bainitic and ferritic-degenereated perlitic grains.

The islands of lower bainite are characterized by augmented dislocation densities leading to the entrapment effect of atomic hydrogen in the crystallographic texture. Additionally, the interfacial regions between the ferritic and bainitic areas are characterized by high misorientation angle distribution factor and provoque the supersaturation of crystal structure in molecular hydrogen and the development of extended microplastic fields. Consequently, these microstructural regions consist the main crack initiation points due to the localized excess of the critical stress intensity factor value. In contrast, the degenerated perlite islands, which are characterized by lower dislocation densities as well as the interfacial regions between the ferritic-perlitic regions, which are characterized by low misorientation angle distribution factors, promote the electrodiffusion process and the propagation of the developed microcrack networks. The microcrack propagation rate increases with the increment of the frequency that the defect coincides the interfaces between the ferritic-perlitic and ferritic-bainitic areas.

**Figure 5:**
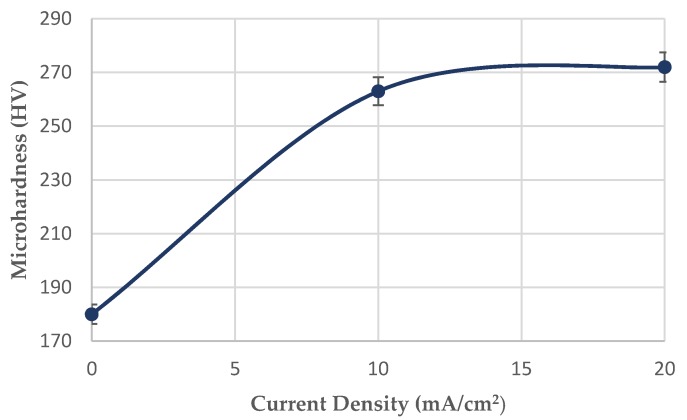
Effect of the applied current density (10 and 20 mA/cm^2^) during the in situ hydrogen cathodic charging process and bending loading on the surface microhardness increment.

After applying the in situ hydrogen cathodic charging process at 10 and 20 mA/cm^2^ current densities, a severe increase in surface hardness was detected (Figure 5). This effect is attributed according to international literature to the interaction between dislocation networks and hydrogen Cottrell atmospheres, to the creation of high volume fraction of hydrogenated vacancies (V-H clusters), and the expanded development of interstitial solid solution [27,28,29,30,31,32,33,34,35]. In particular, the above phenomenon is attributed to the fact that hydrogenated interstisial vacancies of the crystalline structure impede the movement of dislocations, resulting in their interlocking and immobilization effect. The immobilized dislocation networks cannot act as active diffusion channels and the localized supersaturation in hydrogen leads to the accumulation of residual stresses, microplastic zones and slip bands. As a result of the above, the yield stress and the surface micro hardness increase. In any case, the highest rate of the surface microhardness increment was detected during the devolution from the uncharged condition to the cathodically charged under 10 mA/cm^2^ (in comparison to the transition from the charged condition under a current density field of 10 to 20 mA/cm^2^). 

**Figure 6:**
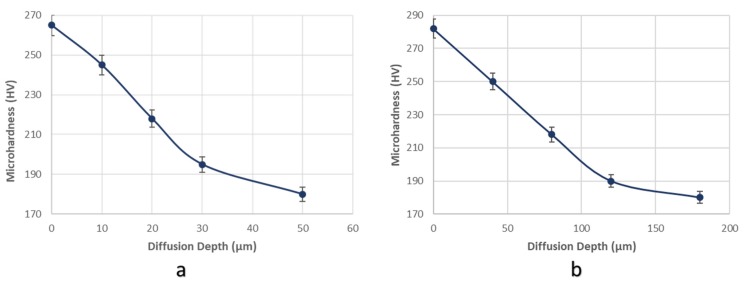
Determination of hydrogen cations’ diffusion depth into the polycrystalline matrix by conducting microhardness mesurements at the cross sections of the specimens, after in situ hydrogen cathodic charging polarization process during slow strain rate bending loading at (**a**) 10 mA/cm^2^ and (**b**) 20 mA/cm^2^ current density.

Additionally, the diffusion depth of hydrogen cations into the microstructure of X65 steel, was validated after in situ hydrogen cathodic charging process by conducting microhardness measurements at the cross section of the specimens. With the increment of the cathodic charging current density from 10 to 20 mA/cm^2^, the diffusion depth of hydrogen cations into the crystal structure augmented from 50 to 180 μm (Figure 6).

By the use of second Fick’s law was calculated the diffusion coefficient of hydrogen cations into the matrix structure.

*D* = *x*^2^/4*t*

where *x*: the hydrogen cations’ diffusion depth (cm) and *t*: the cathodic charging time duration (s).

For a cathodic charging current density field of 10 mA/cm^2^ and in situ hydrogen cathodic charging duration 26 h, the penetration depth of hydrogen cations’ in the matrix region was determined to be 50 µm. Accordingly, the diffusion coefficient was calculated as follows:

*D* = *x*^2^/4*t* = 0.0050^2^ / (4 × 26 × 3600) = 6.67735 × 10^−11^ cm^2^s^−1^

For a 20 mA/cm^2^ cathodic charging current density field and an in situ hydrogen cathodic charging duration 26 h, the penetration depth of hydrogen cations was determined to be 180 µm. Accordingly, the diffusion coefficient was calculated as follows:

*D* = *x*^2^/4*t* = 0.00180^2^ / (4 × 26 × 3600) = 8.65385 × 10^−11^ cm^2^s^−1^

**Figure 7:**
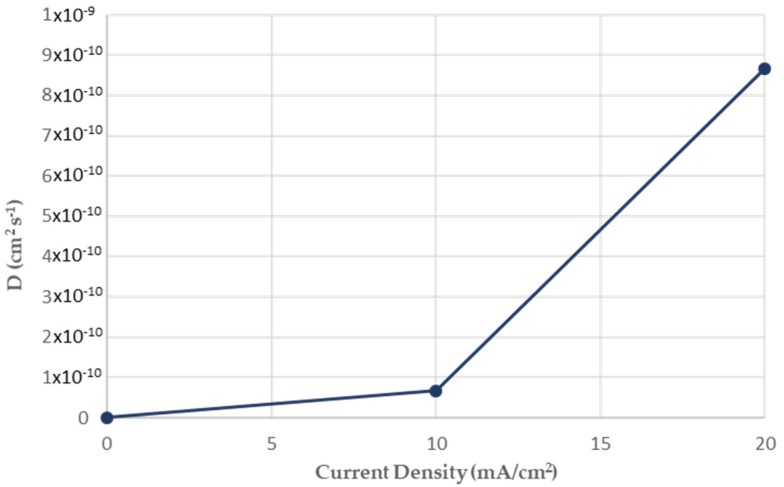
Effect of the applied current density (10 mA/cm^2^ and 20 mA/cm^2^) on the diffusion coefficient of hydrogen cations into the ferritic-bainitic microstructure of X65 pipeline steel.

As it can be seen from Figure 7 the diffusion coefficient of hydrogen atoms into crystalline structure during the in situ hydrogen cathodic charging process and bending loading until fracture was icreased linearly from 6.67735 × 10^−11^ cm^2^s^−1^ to 8.65385 × 10^−10^ cm^2^s^−1^ by increasing the current density field from 10 to 20 mA/cm^2^. This observation can be correlated with the expansion of the electrodiffusion fronts due to the activation of hydrogen cations’ migration effect by easy paths-channels (ferrite-pearlite interfaces) and the excess of the electrostatic-thermodynamic barrier of reversible hydrogen traps (pearlite-bainite interfaces and low angle grain boundaries). 

**Figure 8:**
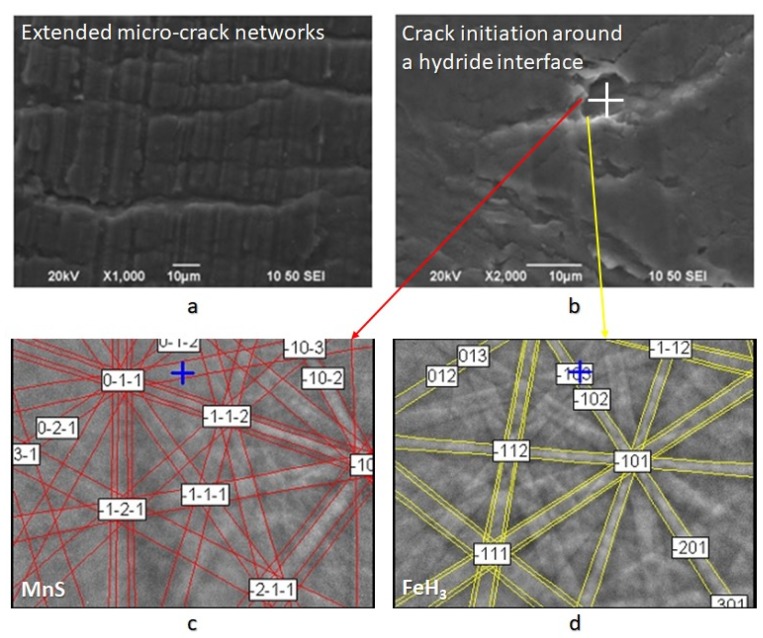
(**a**) Stepwise micro-cracking and micro-cracking branching, (**b**) crack initiation points at hydride/ non-metalic inclusion interfaces, (**c**,**d**) identification of a non metallic inclusion at a high bridge formation from Electron Backscatter Diffraction technique (EBSD).

As it can be seen from Scanning Electron Microscopy images (Figure 8), after the in situ hydrogen cathodic charging process both at 10 and 20 mA/cm^2^, in the surface structure of the X65 pipeline steel was detected micro-cracking phenomenon around the interfaces of blisters and non-metallic inclusions. These micro-cracks propagated by a crooked line morphology (stepwise micro-cracking) as the non-metallic inclusions and the blister formations act like electrokinetic barriers to the development of electrodiffusion process. More precisely these microstructural components act like high-energy irreversible traps and lead to the supersaturation in hydrogen of the matrix material, resulting in the excess of critical stress intensity factor value and the development micro-cracking phenomenon. 

**Figure 9:**
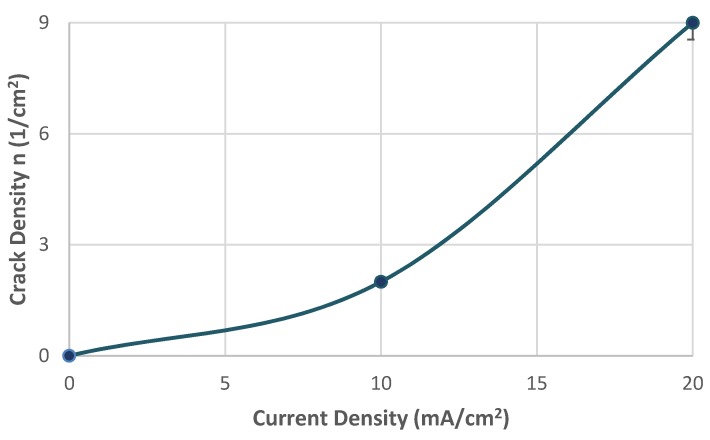
Correlation between surficial density of microcracks and applied current density during in situ hydrogen cathodic charging and slow strain rate bending loading.

As it can be seen from Figure 9 the surficial density of microcracks was observed to increase exponentially (from 2 cracks/cm^2^ to 9 microcracks/cm^2^) with the increment of the required polarization current density during the in situ hydrogen cathodic charging process. The rate of this increase appeared to be higher when switching from 10 to 20 mA/cm^2^ current density compared to switching from the uncharged condition to the charged condition under 10 mA/cm^2^ current density.

**Figure 10:**
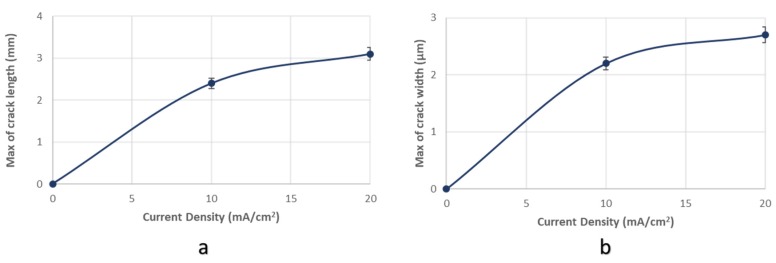
(**a**) maximum crack length and (**b**) maximum crack width of surficial microcracks in conjuction with the applied current density during in situ hydrogen charging process and slow strain rate bending loading.

In addition, the maximum width and maximum length of the microcracks were observed to increase continuously with the increment of the applied current density during cathodic polarization process. For a 10 mA/cm^2^ current density field, the maximum crack width was determined at 2.2 µm and the maximum crack length at 2.4 µm. Correspondingly for a current density field of 20 mA/cm^2^, the maximum crack width was determined at 2.75 µm and the maximum crack length at 3.1 µm. It is, therefore, conceivable that the growth rate of the microcracks is higher during the transition from the uncharged state to the cathodically polarized state under 10 mA/cm^2^ compared to the corresponding one during the transition from the charging process at a current density field of 10 mA/cm^2^ to that of 20 mA/cm^2^ (Figure 10).

**Figure 11:**
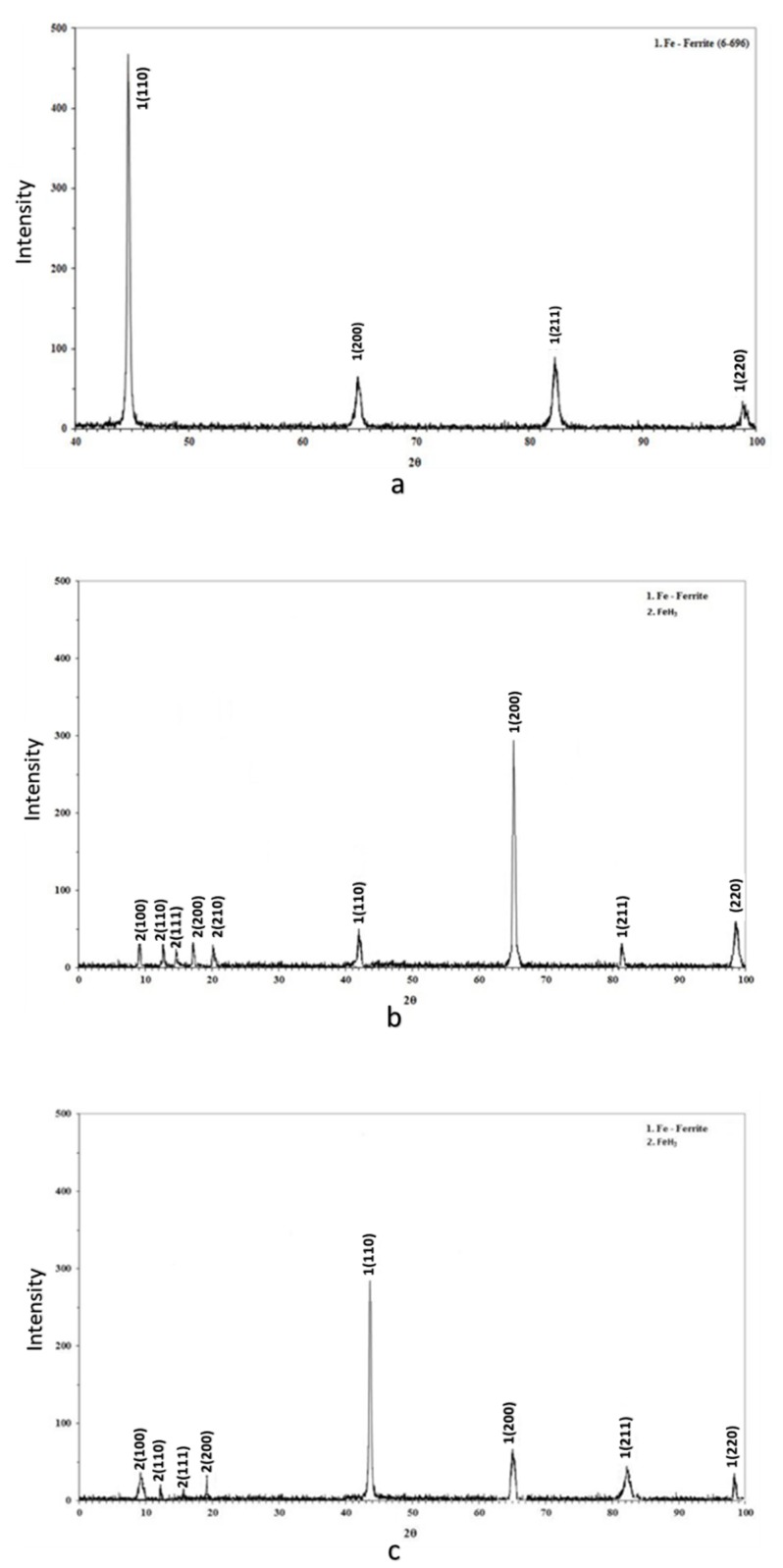
Identification of phases by X-Ray Diffraction patterns (**a**) for the uncharged condition; (**b**,**c**) after cathodic polarization at 10 and 20 mA/cm^2^ current density.

Through X-ray diffractometry, the existence of hydrides with FeH_3_ stoichiometry after in situ cathodic charging process both at 10 and 20 mA/cm^2^ was detected. In addition, the shift of the main diffraction peaks, compared to those of the uncharged specimens, at lower diffraction angles is related to the development of compressive residual stresses within the crystallographic structure during the electrochemical process (Figure 11). 

**Figure 12:**
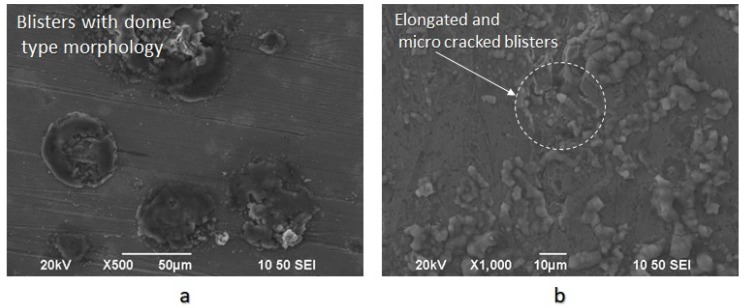
(**a**) Blister formations with a dome-type morphology after the in situ hydrogen cathodic charging process at 10 mA/cm^2^ current density under slow strain rate bending loading; (**b**) Elongated blisters with surficial micro-cracks and blistering on blistering nucleation effect after the in situ hydrogen cathodic charging process at 20 mA/cm^2^ current density under slow strain rate bending loading.

In the surface area of the cathodically charged specimens of X65 pipeline steel, for a current density field of 10 mA/cm^2^, an increased volume fraction of blisters with dome-type morphology was detected. After the polarization process of the specimens at a current density field of 20 mA/cm^2^, the development of elongated blisters was observed, with extended microcrack branching networks at their surface. The microstructural locations correlated with the nucleation process of blisters were the energy upgraded areas, such as the triple junction locations, or the non-metallic inclusions and hydride interfaces (Figure 12) [36,37,38].

**Figure 13:**
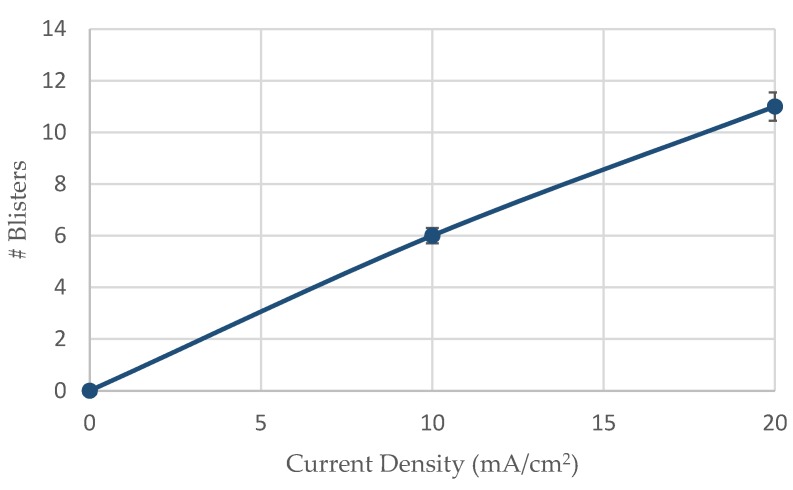
Surficial density of blister formations related to the applied current density during the in situ hydrogen cathodic charging process and slow strain rate bending loading.

As it can be seen from Figure 13 the surficial density of blisters increases proportionally and linearly with the cathodic polarization current density during the electrochemichal charging process. The rate of this increment is observed to be higher during the transition from 10 to 20 mA/cm^2^ current density compared to the transition from the uncharged condition to the polarized condition under 10 mA/cm^2^ current density.

**Figure 14:**
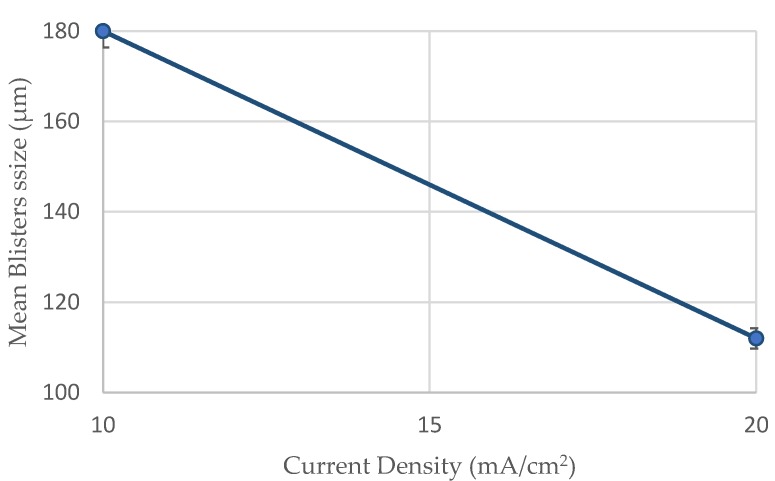
Mean size of blisters developed under the in situ hydrogen cathodic charging process (10 and 20 mA/cm^2^) and slow strain rate bending loading.

Concerning the average growth size of blister formations, it seems to decrease from 180 µm to 110 µm by increasing the required cathodic polarization current density from 10 to 20 mA/cm^2^ (Figure 14). The above observation is attributed to the fact that by the increment of current density field during cathodic polarization process, more nucleation centers for blister formation are activated, thereby limiting their growth rate and mean size.

For the X65 steel specimens subjected to CTOD mechanical testing in ambient air, the mechanical values associated with the fracture toughness parameter were determined as follows: *K*_Q_ = 42.070 MPa·m^1/2^, J = 280.76 KN/m^2^, CTOD_el_ = 0.0061 mm, CTOD_pl_ = 1.92 mm.

**Figure 15:**
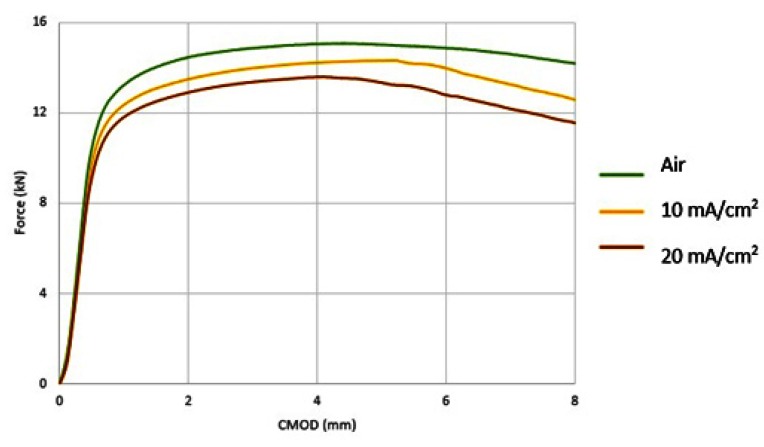
Force-Crack Mouth Open Displacement (CMOD) curves of CTOD tested X65 pipeline steel (**a**) in the uncharged condition; (**b**) after in situ hydrogen cathodic charging at 10 mA/cm^2^ current density; (**c**) after in situ hydrogen cathodic charging at 20 mA/cm^2^ current density.

For the specimens subjected to in situ hydrogen cathodic charging process during slow strain rate bending, a significant decrease in fracture toughness was observed, as it can be seen from the characteristic mechanical values for applied current densities of 10 mA/cm^2^ and 20 mA/cm^2^. More precisely after the in situ hydrogen cathodic charging effect at 10 mA/cm^2^ the main parameters correlated with toughness properties were calculated as follows: *K*_Q_ = 35.04 MPa·m^1/2^, *J* = 202.01 KN/m^2^, CTOD_el_ = 0.0054 mm, CTOD_pl_ =1.44 mm. Respectively after the in situ hydrogen cathodic charging procedure at 20 mA/cm^2^, the above parameters were identified as follows: *K*_Q_ = 30.04 MPa·m^1/2^, *J* = 175.8 KN/m^2^, CTOD_el_ = 0.0037 mm, CTOD_pl_ = 0.98 mm (Figure 15).

Consequently, during the transition from the uncharged state of X65 pipeline steel to the cathodically polarized, for a current density field of 10 mA/cm^2^, the parameters *K*_Q_, *J*, CTOD_el_ and CTOD_pl_ decreased by 16.71%, 28%, 11.48%, 25% respectively. During the transition from the charged condition for a current density field of 10 mA / cm^2^ to that with a current density field of 20 mA/cm^2^, the parameters K_Q_, J, CTOD_el_ and CTOD_pl_ decreased by 28.6%, 37.4%, 39.34%, 48.95% respectively.

**Figure 16:**
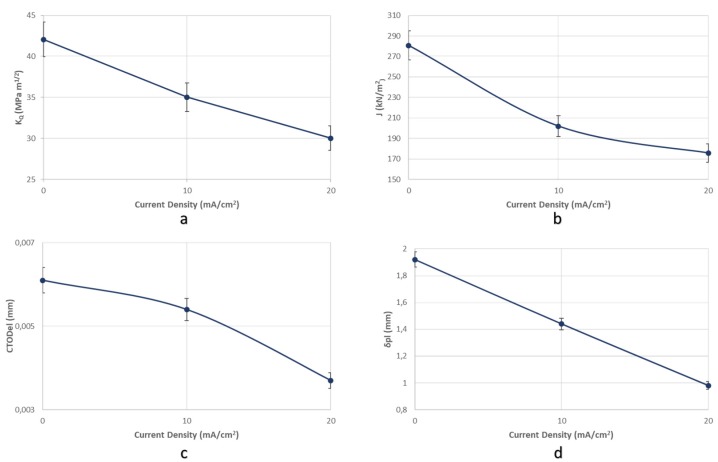
Reduction rate of the main toughness parameters (**a**) *K*_Q_, (**b**) *J*, (**c**) CTOD_el_, and (**d**) CTOD_pl_ in function with the applied current density during the in situ hydrogen cathodic charging process and slow strain rate bending loading.

The rate of the drop of the main toughness parameters was considered to be higher during the transition from 10 to 20 mA/cm^2^ current density, as compared to the transition from the uncharged condition to the cathodically polarized condition under 10 mA/cm^2^ (Figure 16).

The significant drop of the fracture toughness parameters after the in situ hydrogen cathodic charging process of X65 pipeline steel under a current density field of 10 mA/cm^2^, is attributed to the fact that the cathodic polarization procedure causes the supersaturation of surface layers in hydrogen cations, resulting in the development of extensive deformation fields. The consequent work hardening effect of the surface layers is correlated with an elevated microcrack surficial density, which is mainly attributed to the evolution of Hydrogen Enhanced Localized Plasticity (HELP), Hydrogen Induced Cracking (HIC) and Elastic Shielding Reactions mechanisms [39,40,41]. The HELP mechanism is based on the development of highly localized plastic deformation fields, around the hydrogen accumulation points. Moreover, the occurrence of localized plastic deformation fields favors the excess of critical resolved shear stress (τ_CRSS_), at an applied stress much lower than the actual yield strength of the examined material. This results in the material surrounding the localized plasticity zone (that has not yielded yet) to raise a significant barrier to slip action. As a consequence microcracks are developed in the meeting region of the plasticized and the non-deformed material. The HIC mechanism is based on the evolution of high internal pressure inside the matrixes as a result of the extremely high accumulation of hydrogen cations (which exceeds the critical localized chemical potential) [42]. This fact provokes the formation of over-pressurized hydrogen cavities, which lead the surrounding crystal lattice to deform plastically favoring the formation of cracks even without the presence of externally applied load. When the HIC mechanism occurs near the surface of the specimen, it results in the development of blisters as the increased pressure impels the surrounding material to the surface. The latter embrittlement mechanism relies on the limitation of elastic interactions between various stress accumulation fields and structure imperfections, such as dislocation networks and Cottrell hydrogen atmospheres (Elastic shielding of stress centers / H-Shielding) [43]. This limitation is a consequence of the ability of hydrogen atmospheres to reconfigure themselves around stress concentration points and moving dislocations, resulting in the development of severe reaction forces to the overall stress field. For a critical hydrogen concentration barrier within the polycrystalline matrix, the field of repulsion forces tends to be attractive, as a result of the reaction developed due to the extended hydrogen accumulation. The above phenomenon causes dislocation coalescence and favors crack initiation [44].

Under the in situ hydrogen cathodic charging testing with a polarization field of 20 mA/cm^2^, there are two predominant embrittlement mechanisms which provoke this significant deterioration of the mechanical properties. The former is related to the nucleation and development of hydrides (FeH_3_) within the crystalline structure and relies on the fact that hydrides are generally considered to be brittle phases. Therefore the formation of hydrides leads to a considerable reduction in the critical stress intensity factor (KIC) for crack propagation and consequently favors the extended and rapid crack development, causing the drop of the fracture toughness of X65 pipeline steel.

The second one can be attributed to the fact that as the current density field increases during cathodic polarization process, the diffusion coefficient of hydrogen cations into the matrix, the surficial density of microcrack networks and blister formations increases simultaneously, reinforcing the decohesion effect between ferritic and bainitic grains. The responsible embrittlement mechanism for the above observations is the Hydrogen Enhanced Decohesion (HEDE) effect. This phenomenon is based on the fact that atomic hydrogen penetrates into the crystalline structure and occupies intermediate interstitial locations within the crystallographic lattice. In these locations, atomic hydrogen provokes a significant reduction in atomic cohesive energy and strength. As a result, the formation of micro defects is favored, whose enlargement and volume fraction increment catalyze the development of microcracks [44,45,46,47,48].

The fractured surfaces of the specimens that had been submitted under CTOD testing both at the air and hydrogenated environment consisted of the fatigue-precracked area and the bended area.

Specifically for the uncharged and CTOD tested specimens, the fatigue pre-cracked area appeared to consist of benchmark formations, with extended width and intense topographic relief, expanded about 45° from the loading axis (due to the excess of the critical resolved shear stress τ_crss_).

**Figure 17:**
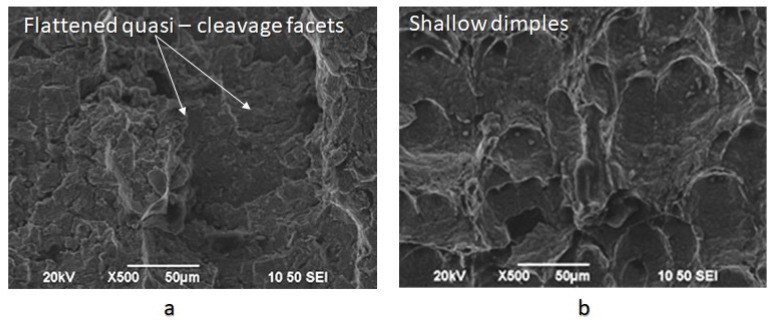
Fractured surfaces after CTOD testing of X65 steel for the uncharged condition (**a**) fatigue pre-cracked area; (**b**) bended area.

The bended area consisted of equiaxial craters with homogeneous distribution and spherical symmetry (Figure 17).

**Figure 18:**
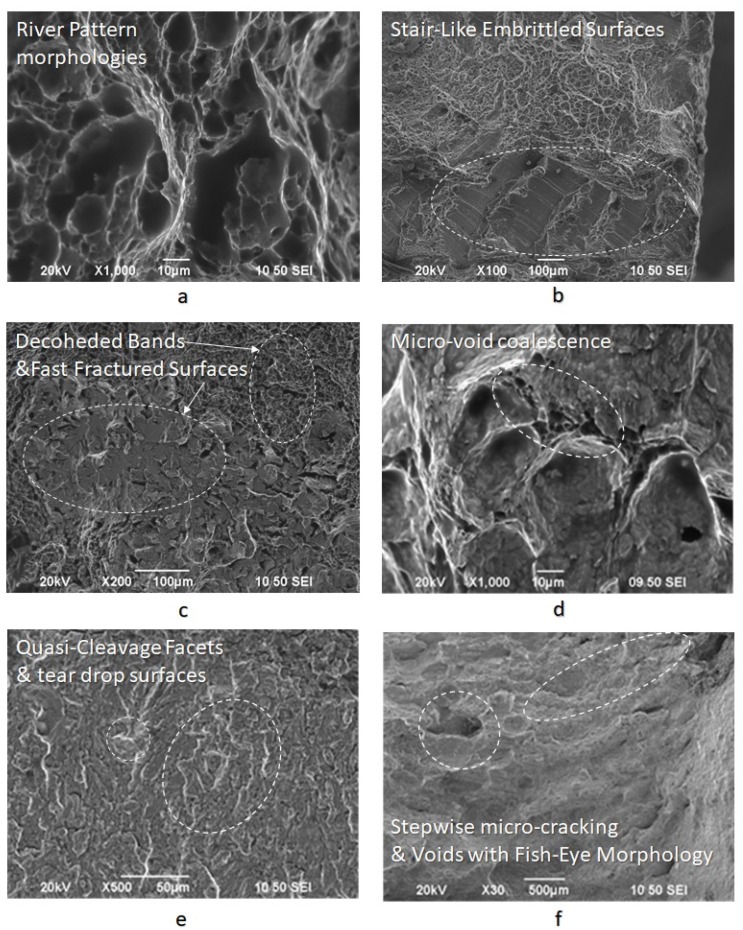
Fractured surfaces after CTOD testing under in situ hydrogen cathodic charging condition at 20 mA/cm^2^, (**a**) river pattern morphologies, (**b**) stair-like embrittled surfaces, (**c**) decoded bands and fracture surfaces, (**d**) micro-void coalescence effect, (**e**) quasi-cleavage facets and teardrop surfaces and (**f**) voids with fish eye morphology and stepwise micro-cracking.

For the specimens subjected to slow strain rate bending loading, under the effect of hydrogen cathodic charging process at a current density field of 10 mA/cm^2^ and for 20 mA/cm^2^, the extensive appearance of embrittled regions, such as quasi-cleavage facets, river pattern morphologies, fast fracture surfaces (FFS), teardrop ridges and stair-like features was detected. The stepwise microcracking phenomenon was also observed simultaneously by the appearance of micro-voids with fish eye morphology due to the evacuation of craters with trapped hydrogen (Figure 18).

The fast fracture surfaces, the tear-drop ridges, and the quasi cleavage facets on the fractured surfaces of the CTOD tested specimens are indicative of the manifestation of the Hydrogen Enhanced Decohesion mechanism (HEDE). Correspondingly, the microvoid coalescence effect and the stair-like/saw-teeth embrittled surfaces confirm the development of Hydrogen Enhanced Localized Plasticity mechanism (HELP) and Elastic shielding reactions phenomenon. Finally, river patterns, micro-voids with fish eye morphology and stepwise micro-cracking are characteristic features of the Hydrogen Embrittlement effect (HE) and are mainly attributed to the mechanisms of Hydrogen Induced Cracking (HIC), Internal Pressure theory (IP) and the development of hydride phases and blister formations.

## 4. Conclusions

The novelty of the results of the present research work is attributed to the use of an environmentally friendly electrolyte (green chemistry), and the imposition of elevated cathodic current densities during the in situ hydrogen cathodic charging process compared to other research works. In addition to the particular electrochemical conditions imposed during the cathodic polarization process of this type of steel, it is the first time that the critical thermodynamic parameters such as hydrogen diffusion coefficient, the penetration depth of hydrogen cations, and surficial density of blisters and microcracks were determined. Finally, for a cathodic current density field of 20 mA/cm^2^, it is the first time that CTOD_el_ and CTOD_pl_ parameters were defined after the in situ hydrogen cathodic charging testing of X65 pipeline steel.

The microstructure of X65 pipeline steel consisted of ferritic and bainitic grains with a high volume volume fraction of degenerated pearlite islands. The average grain size of the ferritic-bainitic microstructure was evaluated by Electron Backscatter Diffraction technique (EBSD) equal to 18 μm.

By the use of X-ray Diffraction and Electron Backscattered Diffraction Technique it was detected the existence into the metal structure of FeH_3_ hydrides, after the in situ hydrogen cathodic charging process, both for 10 and 20 mA/cm^2^ applied current densities.

The islands of lower bainite are characterized by augmented dislocation densities leading to the entrapment effect of atomic hydrogen in the crystallographic texture. Τhe degenerated perlite islands, which are characterized by lower dislocation densities as well as the interfacial regions between the ferritic-perlitic regions, which are characterized by low misorientation angle distribution factors, promote the electrodiffusion process and the propagation of the developed microcrack networks

- After applying the in situ hydrogen cathodic charging process at 10 and 20 mA/cm^2^ current densities, a severe increase in metal surface hardness was detected. This effect is attributed to the interaction between dislocation networks and hydrogen Cottrell atmospheres, to the creation of high volume fraction of hydrogenated vacancies, and the development of interstitial solid solution.
- With the increment of the cathodic charging current density from 10 to 20 mA/cm^2^, the diffusion depth of hydrogen cations into the X65 crystal structure augmented from 50 to 180 μm.
- The diffusion coefficient of hydrogen atoms into the crystalline structure during the in situ hydrogen cathodic charging process and slow strain rate bending loading until fracture was icreased linearly from 6.67735 × 10^−11^ cm^2^s^−1^ to 8.65385 × 10^−10^ cm^2^s^−1^ by increasing the current density field from 10 to 20 mA/cm^2^.
- After the in situ hydrogen cathodic charging process both at 10 and 20 mA/cm^2^ in the surface structure of the X65 pipeline steel was detected the microcracking phenomenon around the interfaces of blisters and non-metallic inclusions.
- The surficial density of microcracks was observed to increase exponentially (from 2 cracks/ cm^2^ to 9 microcracks/cm^2^) with the increment of the required polarization current density during the in situ hydrogen charging process and slow strain rate bending loading. For a 10 mA/cm^2^ current density field, the maximum crack width was determined at 2.2 µm and the maximum crack length at 2.4 µm. Correspondingly for a current density field of 20 mA/cm^2^, the maximum crack width was determined at 2.75 µm and the maximum crack length at 3.1 µm.
- In the surface area of the cathodically charged specimens of X65 pipeline steel for a current density field of 10 mA/cm^2^, an increased volume fraction of blisters with dome-type morphology was detected. After the polarization process of the specimens at a current density field of 20 mA/cm^2^, the development of elongated blisters was observed, with extended microcrack branching networks at their surfaces. Concerning the average growth size of blister formations, it seems to decrease from 180 µm to 110 µm by increasing the required cathodic polarization current density from 10 to 20 mA/cm^2^.
- For the X65 steel specimens subjected to CTOD mechanical testing in ambient air, the mechanical values associated with the fracture toughness were determined as follows: *K*_Q_ = 42.070 MPa·m^1/2^, *J* = 280.76 KN/m^2^, CTOD_el_ = 0.0061 mm, CTOD_pl_ = 1.92 mm. After the in situ hydrogen cathodic charging process at 10 mA/cm^2^ the main parameters correlated with toughness properties were calculated as follows: *K*_Q_ = 35.04 MPa·m^1/2^, *J* = 202.01 KN/m^2^, CTOD_el_ = 0.0054 mm, CTOD_pl_ = 1.44 mm. Respectively after the in situ hydrogen cathodic charging procedure at 20 mA/cm^2^ the above parameters were identified as follows: *K*_Q_ = 30.04 MPa·m^1/2^, *J* = 175.8 KN/m^2^, CTOD_el_ = 0.0037 mm, CTOD_pl_ = 0.98 mm.
- Consequently, during the transition from the uncharged state of X65 pipeline steel to the cathodically polarized under a current density field of 10 mA/cm^2^, the parameters *K*_Q_, J, CTOD_el_ and CTOD_pl_ decreased by 16.71%, 28%, 11.48%, and 25%, respectively. During the transition from the charged condition under a current density field of 10 mA/cm^2^ to that with a current density field of 20 mA/cm^2^, the parameters K_Q_, J, CTOD_el_ and CTOD_pl_ decreased by 28.6%, 37.4%, 39.34%, and 48.95%, respectively.
- The significant drop of the fracture toughness parameters after the in situ hydrogen cathodic charging process of X65 pipeline steel under a current density field of 10 mA/cm^2^, is attributed is related to the contribution of the Hydrogen Enhanced Localized Plasticity (HELP), Hydrogen Induced Cracking (HIC) and Elastic Shielding Reactions mechanisms
- After the in situ hydrogen cathodic charging testing under a polarization field of 20 mA/cm^2^ the significant deterioration of the mechanical properties is attributed to the development of a high volume fraction of FeH_3_ hydrides and the evolution of the Hydrogen Enhanced Decohesion embrittlement mechanism (HEDE).
- The fractured surfaces of the specimens that had been submitted under CTOD testing both at the air and hydrogenated environment consisted of the fatigue-precracked area and the bended area. For the specimens subjected to slow strain rate bending loading under the effect of hydrogen cathodic charging process at a current density of 10 and 20 mA/cm^2^ the extensive appearance of embrittled regions, such as quasi-cleavage facets, river pattern morphologies, fast fracture surfaces (FFS), teardrop ridges and stair-like features was detected.

## Figures and Tables

**Table 1:** Loading characteristics during fatigue precrack stage.

Loading Stage	Cycles	Fmax (kN)	Fmin (kN)	Target Set Point	Amplitude
First stage	9000	−14	−1.4	−7.7	6.3
Second stage	12,000	−10	−1.0	−5.5	4.5
Third stage	50,000	−6	−0.6	−2.7	3.3

**Table 2:** Chemical composition of X65 pipeline steel.

C	Si	Mn	P	S	V	Nb	Ti	Fe
0.16%	0.45%	1.65%	0.02%	0.01%	0.08%	0.05%	0.06%	Bal.

**Table 3:** Mechanical properties of X65 pipeline steel.

Yield Strength Min (KSI)	Tensile Strength Min (KSI)	Yield to Tensile Ratio (max)	Elongation (%)	Hardness (HV)
65	77	0.93	18	220
